# Amyloid beta oligomers induce neuronal elasticity changes in age-dependent manner: a force spectroscopy study on living hippocampal neurons

**DOI:** 10.1038/srep25841

**Published:** 2016-05-13

**Authors:** Andreea-Alexandra Ungureanu, Iryna Benilova, Olga Krylychkina, Dries Braeken, Bart De Strooper, Chris Van Haesendonck, Carlos G. Dotti, Carmen Bartic

**Affiliations:** 1 Department of Physics and Astronomy, KU Leuven, Celestijnenlaan 200D, B-3001, Leuven, Belgium; 2 imec, Kapeldreef 75, B-3001 Leuven, Belgium; 3 VIB Center for the Biology of Diseases, ON 4 Campus Gasthuisberg, Herestraat 49, B-3001, Leuven, Belgium; 4 CSIC, Centro de Biología Molecular Severo Ochoa, Universidad Autónoma de Madrid Campus Cantoblanco, 28049 Madrid, Spain

## Abstract

Small soluble species of amyloid-beta (Aβ) formed during early peptide aggregation stages are responsible for several neurotoxic mechanisms relevant to the pathology of Alzheimer’s disease (AD), although their interaction with the neuronal membrane is not completely understood. This study quantifies the changes in the neuronal membrane elasticity induced by treatment with the two most common Aβ isoforms found in AD brains: Aβ40 and Aβ42. Using quantitative atomic force microscopy (AFM), we measured for the first time the static elastic modulus of living primary hippocampal neurons treated with pre-aggregated Aβ40 and Aβ42 soluble species. Our AFM results demonstrate changes in the elasticity of young, mature and aged neurons treated for a short time with the two Aβ species pre-aggregated for 2 hours. Neurons aging under stress conditions, showing aging hallmarks, are the most susceptible to amyloid binding and show the largest decrease in membrane stiffness upon Aβ treatment. Membrane stiffness defines the way in which cells respond to mechanical forces in their environment and has been shown to be important for processes such as gene expression, ion-channel gating and neurotransmitter vesicle transport. Thus, one can expect that changes in neuronal membrane elasticity might directly induce functional changes related to neurodegeneration.

Alzheimer’s disease (AD) is an age-associated neurodegenerative disorder correlated with the abnormal production and aggregation of small amyloid-beta (Aβ) peptide species^1^. Various studies have shown that aggregated Aβ species impair synaptic transmission and initiate apoptosis via several mechanisms, such as accumulation of intracellular Ca^2+^ through pore formation, disruption of the lipid membrane through a detergent-like effect, hyperphosphorylation of tau protein, and activation of caspase-3^2^,^3^.

Aβ peptides are generated by the proteolytic processing of the transmembrane protein Amyloid Precursor Protein (APP) into isoforms with different numbers of amino acids at the C terminus^4^. The most common Aβ isoforms found in AD brains are peptides with 40 (Aβ40) and 42 residues (Aβ42)^5^. The amphiphilic nature of the Aβ peptides plays an important role in the aggregation of stable or metastable intermediary species, such as soluble oligomers, protofibers, annular aggregates and fibrillar species^6^. The two extra hydrophobic residues at the C-terminus of the Aβ42 variant influence its aggregation behavior, resulting in the formation of more stable β-sheet structures by Aβ42 than Aβ40^7^. Amyloid fiber formation is conditioned by an initial nucleation–growth mechanism that has a lag-phase with different durations for Aβ40 and Aβ42. Several studies have indicated that differences in the aggregation behavior are responsible for the differences in the neuronal toxicity of the Aβ40 and Aβ42 peptides. Aβ40 has a slower aggregation rate and has been shown to be less neurotoxic, whereas the more hydrophobic Aβ42 has faster aggregation kinetics and forms more toxic oligomeric species^8^,^9^,^10^. A growing body of evidence indicates that the neurotoxic species formed by Aβ peptides are the small oligomers occurring in the initial aggregation lag phase and are not monomers or fibers^8^,^11^. Model lipid bilayer systems have revealed that Aβ monomers have nearly no effect on the lipid bilayer, whereas oligomeric species alter the physicochemical properties of lipid bilayer systems^12^,^13^,^14^,^15^,^16^.

Amyloid-beta peptide interactions with cellular membranes are highly complex and depend on the interplay between the properties of the peptides and those of their physico-chemical environment, including the membrane^17^. The chemical and mechanical properties of cellular membranes are age-dependent. The specific ratio of the lipid components, i.e., cholesterol, sphingomyelin, gangliosides^18^, as well as their charge, were found to influence the affinity of Aβ species for the neuronal membrane^2^,^11^,^19^ and their insertion into the membrane. For instance, Aβ species specifically bind to GM1 gangliosides, depending on the presence of cholesterol, forming an Aβ/GM1 complex^20^,^21^,^22^. Positively charged amino acids, such as Arg5, Lys16 and Lys28 present in the peptide structure facilitate the attachment of Aβ to negatively charged lipids, such as phosphatidylserine and phosphatidylglycerol. Cholesterol is an important component of the cellular membrane and dictates membrane fluidity as well as the density, stability and organization of lipid rafts^23^. Several studies have suggested that the affinity and insertion ability of Aβ in the lipid bilayer is controlled by the lipid composition of the membrane, with cholesterol being a key player^18^,^24^,^25^.

Thus, age-related changes in the lipid composition of the neuronal membrane appear to be important for Aβ production and neurotoxicity^26^,^27^,^28^,^29^. McKee and colleagues showed that Aβ had nearly no effect on the brains of young primates but initiated a neurotoxic cascade in the brains of aged primates^30^. Aging of hippocampal neurons is accompanied by a decrease in the cholesterol level in the cellular membrane as shown in both human^31^ and rodent studies^32^.

In this study, we investigated the mechanical properties of primary hippocampal neurons treated with Aβ oligomeric species. More specifically, using quantitative atomic force microscopy (AFM), we measured the changes in the elastic properties of the neuronal membrane induced by treatment with Aβ40 and Aβ42 peptides in the initial aggregation stage (i.e., after 2 hours of peptide aggregation). We selected this particular aggregation time window because previous studies showed that the exposure of cultured primary hippocampal neurons to a 2 hours pre-aggregated Aβ42 solution has a strong neurotoxic effect^8^. More specifically neuronal activity recordings using Multi Electrode Arrays (MEAs) revealed a dramatic decrease in spontaneous electrical activity of neurons treated with 2 hours - aggregated 10 μM Aβ42 oligomer solution (i.e. ~25% activity decrease after 5 minutes of exposure and ~100% after 20 minutes of exposure – see Supplementary Information). No significant activity changes were detected in the case of Aβ40 exposure.

In terms of cellular viability, no significant viability changes are occurring when the duration of the Aβ treatment is less than 4 hours. In the case of longer treatments with Aβ42, the neuronal viability is reduced (i.e. we measured a viability drop of about 60% after 24 hours treatment with 2 hours aggregated Aβ42 10 μM solution).

In this study we addressed the effects of age and aging conditions on the mechanical properties of neurons treated with the two Aβ species under pre-lethal conditions, i.e. before significant viability changes start to occur. Otherwise, major changes in the membrane mechanical properties would result as direct effect of necrotic processes. Therefore we investigated the elastic properties for Aβ exposure times below 4 hours.

Neurons were cultured for 1 week and 3 weeks *in vitro* under two different sets of culturing conditions. In a medium mimicking physiological conditions (i.e., Primary Neuron Growth medium (PNGM)), we obtained fully developed young neurons after 7 days *in vitro* (DIV) (hereafter referred to as 1wPNG). 21 DIV growth under the same conditions allowed the neurons to develop in an environment rich in vitamins and antioxidants. We refer to neurons of this type as mature neurons (or 3wPNG). A third neuronal preparation was obtained in a medium that lacked neurotrophic factors, vitamins and antioxidants (i.e., Neurobasal plus N2 supplement medium (NBN2)). The neurons developed in this medium displayed enhanced stress/aging hallmarks, such as larger lipofuscin aggregates and reduced membrane cholesterol content. This neuronal preparation is hereafter referred to as “aged” (or 3wNBN2).

Using the force spectroscopy mode of the AFM, we measured changes in the static elastic modulus of these three neuronal preparations (i.e., young (1wPNG), mature (3wPNG) and aged (3wNBN2) neurons) when exposed to the Aβ40 and Aβ42 soluble species formed after 2 hours of aggregation of the Aβ monomeric solution prior to neuronal treatment at room temperature (RT).

AFM techniques, with theoretical nanometer-size resolution, have been applied in different modalities to investigate the aggregation of Aβ peptides and their interaction with reconstituted bilayers. AFM studies revealed the morphology and distribution of aggregated species and have demonstrated the insertion and pore formation in synthetic bilayers^33^,^34^,^35^. In addition, force spectroscopy has been used to evaluate the elastic modulus of different neuron types^36^. Nevertheless, AFM studies on living hippocampal neurons have been rare, given the extremely soft and sensitive nature of these cells (which have static elastic modulus values of less than or equal to 100 Pa). Working with weak applied forces is crucial in imaging such preparations to avoid cellular stress and injury, and this requires exquisite force control and low noise in the AFM system, as well as a large number of measurements to increase data reliability.

An earlier AFM study based on single cell compression experiments proposed that the insertion of Aβ42 in the membrane alters the mechanical properties of stable cell lines^37^. N2a and HT22 cell lines treated with Aβ42 oligomers were deformed between a flat glass slide and a spherical indenter up to 80% of the cellular height, and the required force was measured. These results showed that cells treated with Aβ42 prefibrillar oligomers were able to sustain a much higher deformation force (i.e., a significant cellular stiffening was measured), which the authors attributed to an increase in osmotic pressure via unregulated ion flux due to Aβ42 insertion^37^.

In this study, however, we probed the elastic properties of primary hippocampal neurons in the low indentation force regime. Indentation forces below 1 nN (inducing deformations smaller than 10% of the cellular height) were used to probe the elastic modulus of the soma and ensure that no damage is inflicted and that the conditions for elastic deformation are being satisfied. We show that relatively short incubation periods (i.e. 1–4 hours, see below) of the hippocampal neuron preparations described above with Aβ40 and Aβ42 oligomers induce neuronal elasticity changes in an age-dependent way, before significant cell damage by the Aβ42 oligomers was inflicted. The effect of neuronal age on the amyloid-beta/membrane interactions was revealed by changes in the elastic modulus of the membrane/actin cortex upon amyloid treatment. Aged neurons were the most susceptible to Aβ binding and showed the largest membrane elasticity change after treatment with both Aβ40 and Aβ42.

Our results show that the species formed by the 2-hours aggregation of Aβ40 and Aβ42 have different effects on intact young and aged neurons, with the aged neurons undergoing larger changes in the elastic properties than the young neurons. Both peptide species interact strongly with the membrane of aged neurons and dramatically reduce membrane stiffness, suggesting a relatively non-specific membrane interaction (possibly a disruption of the lipid bilayer). Because mechanical forces are known to play an important role in processes such as gene expression, ion-channel gating and vesicular transport, changes in membrane elasticity may also be directly correlated with functional abnormalities linked to neurodegeneration. For instance, membrane tension has been shown to affect ion-channel activities^38^ and vesicular transport in synapses^39^ as well as other functions^40^. Our AFM data indicate that neurons aged under stress conditions undergo a significant membrane softening process when exposed to Aβ oligomers under non-lethal conditions.

## Results

### Characterization of Aβ40 and Aβ42 species pre-aggregated in solution

Prior to evaluating the neuronal responses to Aβ40 and Aβ42 oligomers, we characterized the species formed by these peptides after several hours of aggregation in Tris-EDTA (Trisaminomethane - Ethylene-Diamine-Tetraacetic Acid) buffer. The two peptides have previously been reported to have different aggregation behaviors, as determined using Thioflavin T (ThT) measurements, AFM snap-shots and theoretical calculations^8^,^41^,^42^. Moreover, our previous *in vitro* and *in vivo* studies have shown that the smaller species formed by Aβ42 are more efficient at triggering toxic mechanisms in neurons than Aβ40^8^. However, the aggregated species obtained in different studies are strongly dependent on the preparation methods used. In this study, we followed a three-step protocol that includes a desalting column and ensures a biocompatible, solvent-free Aβ monomeric solution in Tris-EDTA buffer^43^. The desalting column step ensures that the Aβ monomeric solution is DMSO-free. This is important because the presence of the DMSO solvent could further affect the Aβ aggregation process^44^ and induce toxic effects^45^ and/or membrane solubilization^46^. Our previous studies showed that incubating the Aβ42 monomeric solution at room temperature (RT) for 2 hours resulted in the formation of small soluble oligomeric species^8^. The species formed by Aβ42 under these aggregation conditions trigger neurotoxicity, as demonstrated by the significant reduction of the neuronal activity after only 5 minutes of exposure as recorded in Multi Electrode Arrays (MEAs)^8^ (see Fig. S1 - Supplementary Information).

**Figure 1:**
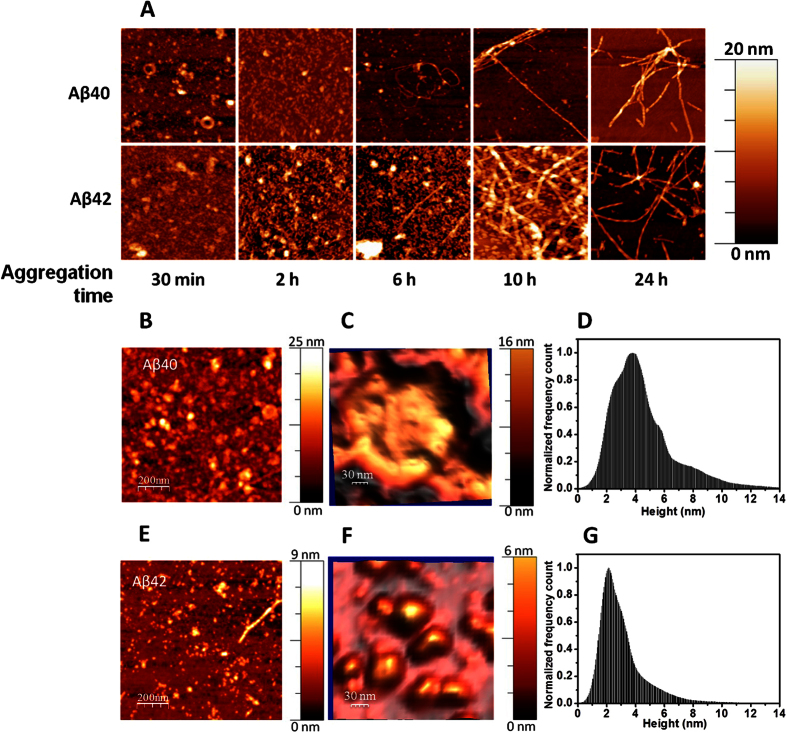
(**A**) AFM images (1 μm × 1 μm) of Aβ40 and Aβ42 species formed in solvent free Aβ monomeric solution in Tris EDTA buffer, aggregated at room temperature for different time periods −30 minutes, 2 hours, 6 hours, 10 hours, and 24 hours and deposited on clean SiO_2_ substrates; (**B**–**D**) Aβ40 forms oligomers with a height ranging between 1 and 5 nm, and these coexist with larger aggregates with heights of approximately 10 nm; (**E**–**G**) Aβ42 forms predominantly lower molecular weight oligomers with a height ranging between 0.5 and 2 nm, coexisting with protofibers with heights between 2 and 5 nm. A few fibers were formed by both peptides after 2 hours. Quantitative analysis of the AFM data in (**D**,**G**) was performed by averaging the height histograms from eight images for Aβ40 and 16 images for Aβ42 (1 μm × 1 μm area scans).

Figure 1A summarizes the results of the AFM morphological characterization of the Aβ40 and Aβ42 species formed after aggregation periods of up to 24 hours, as deposited onto a clean SiO_2_ surface.

As shown in Fig. 1B–G, after 2 hours of aggregation, the smallest oligomeric species (height ranging between 0.5 and 2 nm) were detected for Aβ42, and these species coexisted with protofibers with heights between 2 and 5 nm and a small number of larger aggregates (including fibers). Aβ40 forms amorphous oligomeric aggregates with height values ranging between 1 and 10 nm. The height histograms are shown in Fig. 1D,G. In both cases, fibers are already present after 2 hours of aggregation (although less abundant), demonstrating that both peptides could complete the fibrillization pathway in such a short period of time. The coexistence of oligomeric, prefibrillar and fibrillar species with different sizes indicates a non-linear aggregation behavior of the Aβ peptides, consistent with findings obtained in other studies^47^,^48^. It is important to note that AFM imaging of surface-deposited amyloid species predominantly reveals the smallest present species since larger aggregates are less adsorbing onto the silica surface. When analyzing the size distribution of the amyloid species in solution using dynamic light scattering (DLS), a more heterogeneous size distribution was detected for Aβ42 than for Aβ40, with still the smallest aggregate sizes measured for Aβ42 (see Fig. S2 in Supplementary information for the DLS size distributions).

To conclude, smallest oligomeric species (height between 0.5 and 2 nm) are present in the 2 hours aggregated Aβ42 solutions.

### Properties of Aβ40- and Aβ42-treated neurons

AD has a high prevalence in the elderly population, which raises the question about the susceptibility of the aged brain to toxic Aβ peptides^49^. Usually, studies of Aβ neuronal effects are performed for a single population age of neurons. To investigate whether neuronal aging and aging conditions are influencing the Aβ affinity and insertion, we prepared hippocampal neuron cultures following three different protocols and obtained young (1 week = 7 DIV), mature (3 weeks  = 21 DIV) neurons cultured in PNG medium and aged neurons (21 DIV culture in a medium deprived of antioxidants and trophic factors - NBN2 medium). In the last condition, the culture medium consisted of Neurobasal medium with a N2 supplement (NBN2), which is able to maintain the viability of the neurons but accelerates the aging process due to the absence of trophic factors^50^.

Aging post-mitotic cells, such as neurons, are associated with intracellular accumulation of metabolic by-products, such as reactive oxygen species (ROS) and lipofuscin vesicles, which might interfere with cell survival. Several studies have shown that in normal aging, post-mitotic cells accumulate lipofuscin in a linear manner^51^. This autofluorescent pigment can be used as an aging marker, permitting the estimation of long-term oxidative stress accumulated in neurons^32^.

**Figure 2:**
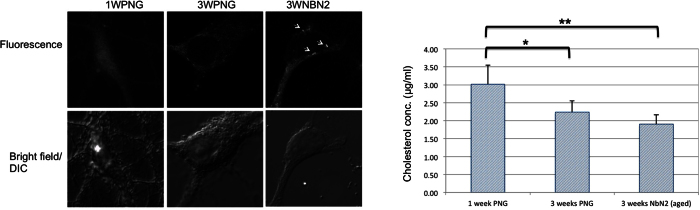
(**A**) Fluorescence images (upper panels) showing increasingly larger autofluorescent aggregates attributed to lipofuscin vesicles in 3 neuronal preparations; 3 week old neurons cultured in the NBN2 medium (3^rd^ columns) exhibit the largest aggregates. Each image size is 35 × 35 μm^2^. (**B**) Cholesterol concentration in the membrane pellets of hippocampal neurons at different stages of aging: 30% lower cholesterol levels were detected for 3-week-old neurons cultured in PNG compared to 1-week-old cells. Neurons cultured in NBN2 showed a more pronounced decrease in membrane cholesterol levels. *p = 0.1 for at least 9 independent data points per sample **p = 0.02 (unpaired Student t-test) for at least 7 data points per sample. Error bars represent the standard error of the mean for each sample.

Fluorescence images of young hippocampal neurons (7DIV) reveal uniform autofluorescence without lipofuscin accumulation. Longer incubation in PNG media allows the neurons (3wPNG) to mature and form more complex connections with almost no lipofuscin vesicles visible in the cytoplasm. In the case of 21DIV neurons cultured in NBN2 medium (3wNBN2), large aggregates of lipofuscin vesicles can be detected as a marker of an advanced stress/aging stage as shown in Fig. 2A.

We also measured different cholesterol concentrations in the membrane pellets obtained from the three neuronal types used in this work: young (1wPNG), mature (3wPNG) and aged (3wNBN2). As shown in Fig. 2B, there is a significant difference in cholesterol levels between young and 3-week-old neurons. Mature neuronal membranes present about 30% less cholesterol than young neurons, consistent with the findings obtained in other literature studies^26^,^31^. Neurons deprived of antioxidants and growth factors (i.e., cultured in NBN2 medium), have slightly lower membrane cholesterol contents than neurons cultured in PNG medium. Although cholesterol has been proposed to play an important role in amyloid insertion into the cell membrane, for the purposes of the mechanical property investigation described here, the decrease in the cholesterol level was only used to characterize the aging process.

We employed fluorescence microscopy to assess the difference in Aβ susceptibility of the neurons at three different stages of aging. As described in Kuperstein *et al.*^8^, previous studies showed that pure Aβ42 peptides produce neurotoxic oligomers when aggregated for several hours at RT prior to the neuronal treatment. The highest neurotoxic effect was induced by the Aβ42 oligomers after 2 hours of aggregation, whereas no neurotoxicity was observed for Aβ40 oligomers aggregated under similar conditions. The effect of Aβ42 on the neuronal firing rate (i.e. decrease in the firing rate) was detectable after only 5 minutes after the addition of the 2 hours aggregated Aβ42 to the neuronal culture and increased with the exposure time. The activity was completely suppressed after 30 minutes of exposure (see Supplementary Information).

**Figure 3:**
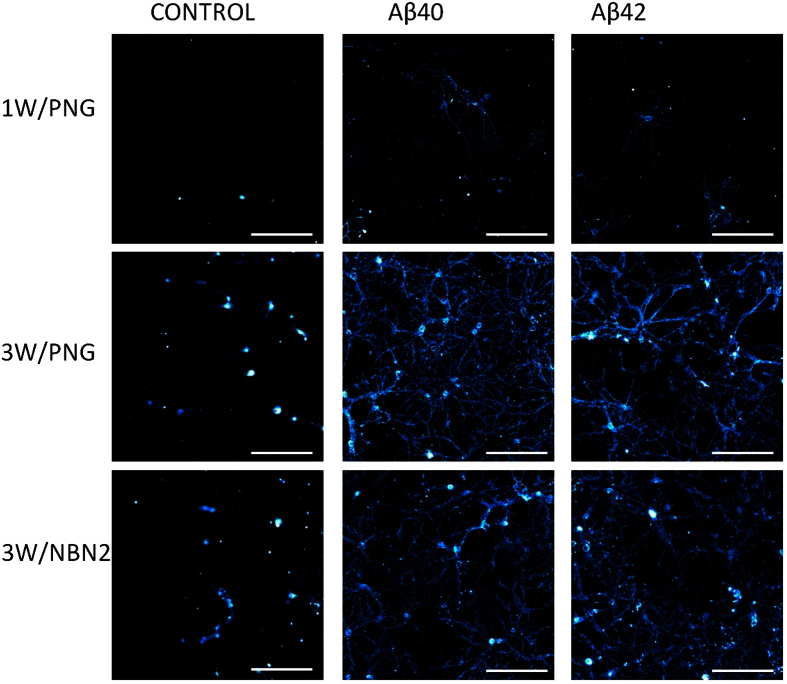
Epifluorescence images showing the different susceptibilities of hippocampal neurons to Aβ oligomers (i.e., Aβ-positive neurons). Stainings with Beta Amyloid 1–16 (6E10) Monoclonal Antibody, Alexa Fluor 488: Labeled young neurons (1W/PNG – upper row), mature neurons (3W/PNG – middle row) and aged neurons (3W/NBN2 – lower row); scale bars are 20 μm.

Therefore we comparatively investigated the effects of Aβ40 and Aβ42 aggregated using the same protocol on the mechanical properties of the neurons. The cultures were incubated for 1 hour in 10 μM Aβ40 or Aβ42 pre-aggregated solutions prior to the start of the AFM measurements. Since the AFM measurements are time consuming (i.e. one measurement session can take up to 5 hours), the different cells probed by AFM were exposed to the Aβ pre-aggregated solutions for time intervals between 1 and 6 hours. Therefore for the fluorescence assessment of the neuronal susceptibilities, we treated the neuronal preparations for 3 hours (as an average exposure time relevant for the AFM probing) with 10 μM solutions of pre-aggregated Aβ40 or Aβ42. The Aβ-treated neurons were fixed and stained with an anti-Aβ fluorescent primary antibody: Beta Amyloid 1–16 (6E10) Monoclonal Antibody, Alexa Fluor 488 Labeled. The 6E10 primary antibody recognizes amino acids 1–16 of the N-terminus region of the Aβ peptide, thus binding to both Aβ40 and Aβ42 peptides. Overlaying bright field and fluorescence images of Aβ-treated cells revealed that not all neurons were positive for anti-Aβ staining, and that the different preparations had different affinities for the two peptides. Images in Fig. 3 reveal punctate staining of the neuronal soma and processes. The percentages of Aβ-positive neurons to total neuron number in the imaging field are summarized in Table 1.

Both peptides bind poorly to young neurons compared to older neurons: approximately half of the young neurons were Aβ42-positive, compared to about 34% Aβ40-positive (see Table 1). 80% of the mature neurons were positive for both peptides. 3-week-old neurons deprived of antioxidants and growth factors (3wNBN2) showed an even higher affinity (>90%) for both Aβ species. From these data, we can conclude that the Aβ soluble species attach more efficiently to the membrane of old neurons than to those of young cells, whereas no significant differences were found between neurons of the same age in different media.

As shown by the coefficient of variation (CV) in Table 1, the susceptibility of young neurons (1wPNG) to Aβ40 was highly variable (CV = 73%), and it became more uniform with age (~5% for aged neurons). Remarkably, although a similar trend was observed for Aβ42, the variability was significantly lower, indicating that Aβ42 binds to all neuronal preparations more efficiently than Aβ40, with the aged neuronal preparation (3wNbN2) being the most susceptible.

**Figure 4:**
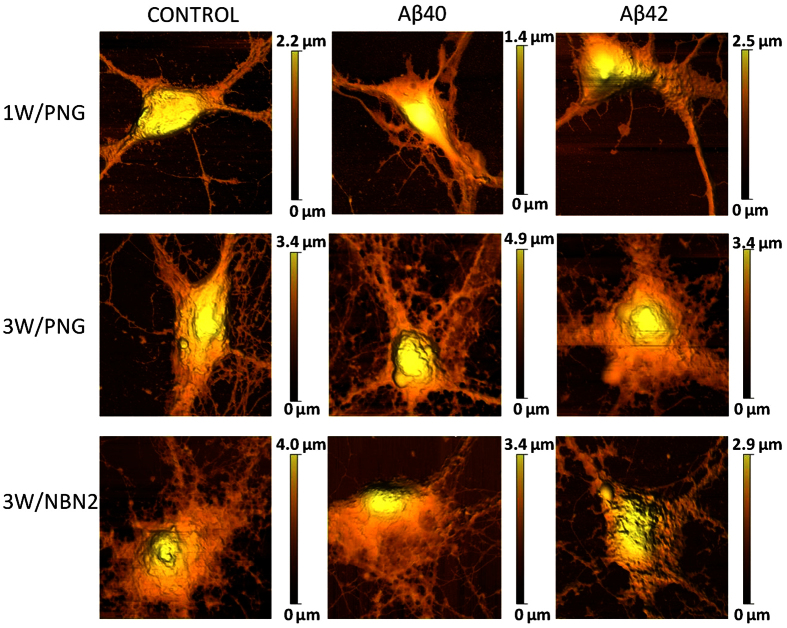
Topographic AFM images of fixed neurons with and without Aβ treatment. Image sizes are 30 μm × 30 μm and were performed on fixed neurons using a sharp pyramidal probe attached to the MSNL-F cantilever.

To evaluate potential morphological changes induced by Aβ treatment, AFM topography mapping was performed on control and Aβ-treated neurons. Imaging the high aspect ratio neuronal somas represents a challenge for AFM topographic scanning, particularly in the classic contact or tapping modes. A solution is provided by the AFM Quantitative Imaging mode, which uses force-distance data to retrieve topographical information. The disadvantage of this method is that high-resolution imaging is very slow: for instance, a scan of 256 × 256 pixels takes approximately 2 hours. Therefore imaging of living neurons at this resolution is almost impossible. Because neurons are sensitive cells that react rapidly to stress factors, and since we were aiming for high-resolution imaging, we performed the topographical scans on fixed neurons. Representative AFM topographical images of neurons fixed after the 3 hours exposure to soluble Aβ species are shown in Fig. 4. Although the fixation procedures may in principle introduce more morphological changes than the Aβ treatments, the obtained AFM images are showing that the 3 hours Aβ exposure does not appear to dramatically influence the morphology of neurons. Only subtle differences are visible, particularly for aged neurons treated with Aβ42. In this case, the neuritis network appears more fragmented and the neuronal filopodia seem more retracted than the control condition. Confocal fluorescence imaging of the same cells (data not shown) cannot resolve the cellular morphology at this level of detail. Based on these results we can conclude that the 3 hours exposure to the 2 hours pre-aggregated Aβ species is not producing detectable changes at this imaging resolution. This is in line with the results of the cell viability tests showing that Aβ exposure of neuronal cultures does not induce significant changes in the viability for exposure times below 4 hours, although the viability is significantly decreasing for longer Aβ42 exposure times (~24 hours) (see Fig. S3 - Supplementary information).

### AFM membrane elasticity study

Force spectroscopy data acquired on Aβ-treated living neurons revealed different effects of the 2 hours pre-aggregated amyloids on the different neuronal preparations. All the measurements were performed on living neurons under physiological conditions (i.e. PNG or NBN2 media, and constant temperature, 37 °C) in the JPK Bio-Cell sample holder.

Prior to the AFM elasticity measurements, the neuronal cultures were incubated for 1 hour at 37 °C with 10 μM solutions of Aβ40 or Aβ42 (pre-aggregated for 2 hours in their respective culture media) prior to performing the elasticity measurements.

The mechanical properties of the neuronal cell body were measured by single cell indentations atop the nucleus. The shape and size of the indenter permits a uniform distribution of the applied force on the neuronal soma, thereby limiting the risk of membrane damage. Given the large contact area between the spherical probe and soma, the cellular static elastic modulus can be measured^52^.

**Figure 5:**
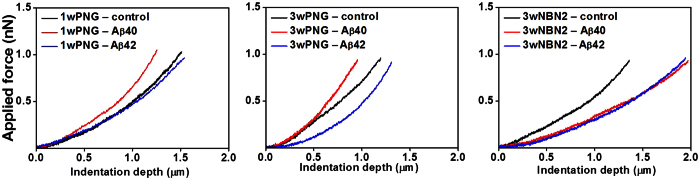
Examples of force-indentation curves recorded for 1wPNG, 3wPNG and 3wNBN2 pyramidal neurons; constant ramping speed of 5 μm/s, with and without Aβ treatments.

Figure 5 displays representative force-indentation curves recorded for a set point of 1 nN with a constant ramping speed of 5 μm/s for the three neuronal preparations treated with Aβ40 and Aβ42. The force-indentation curves of the different preparations without Aβ treatment were similar.

The Young’s moduli of the cells were calculated by fitting the experimental force-indentation curves with the Hertz model adapted for a spherical indenter in the JPK data processing software. The Hertz model has been widely used to extract the Young’s modulus of a cell from the AFM force spectroscopy data^53^. The model is valid under the assumptions that a cell is a homogeneous elastic medium and that the indenter is non-deformable^52^. The first assumption is reasonable only for very small deformations: i.e., the indentation depth should be less than 10% of the sample thickness. We ensured that this condition is valid by limiting the applied force (set point) such that the indentation is maintained below 10% of the cellular height. As shown in Fig. 5, a force of 0.5 nN may induce a 1 μm indentation, which is already ~25% of the total cell height. Thus, in order to extract the Young’s moduli we recorded the force-indentation curves for lower set point values resulting in indentation depths smaller than 500 nm.

The second condition is ensured by the higher stiffness of the indenter compared to the cell (as Young’s modulus of the polystyrene bead is approximately 20 times larger than the cell modulus).

**Figure 6:**
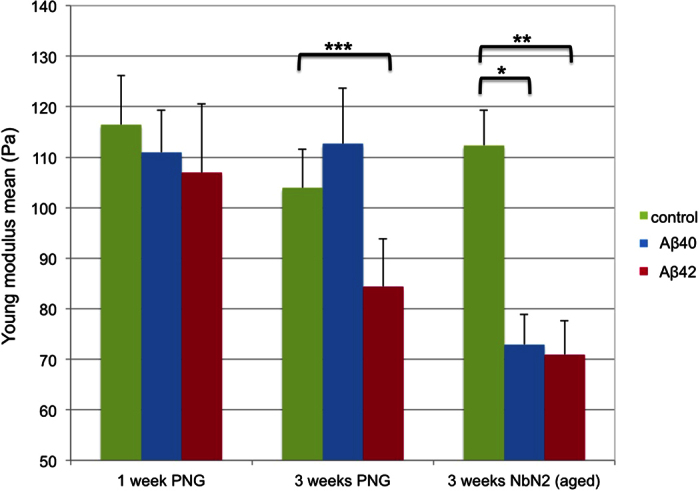
Young’s modulus mean values obtained from AFM measurements on neurons in different development stages (young, mature and aged) treated with Aβ40 or Aβ42. Shallow deformation (below 0.2 nN) by a 15 μm spherical probe of the neuron soma produce indentations between 0.2 and 0.6 μm (less than 10% of the cell height). This condition is important for the validity of the Hertz model. Untreated neurons under different aging conditions have similar elasticity mean values, approximately 100 Pa, as calculated from the AFM force-indentation curves. The culture conditions appear to significantly alter the effect of Aβ on membrane elasticity. For 1- and 3-week-old neurons incubated in PNG media Aβ oligomers had different effects. Aβ42 softened the membrane in all neuronal preparations. The Young’s modulus of aged neurons incubated in NBN2 media was dramatically affected by both Aβ species, presenting a decrease of 30%. *p, **p < 0.0001, ***p = 0.06 by unpaired Student t-test) (each sample had more than 30 points). Error bars represent the standard error of the mean for each condition.

Data on the elastic properties of living cells usually display a large spreading from one cell type to another, or between different cells within the same culture, or from one culture to another and are strongly dependent on the measurement conditions (e.g. indenter shape and size, indentation force and speed, and even the indentation history)^36^. The large data spreading is due to the variability in the cell morphologies (e.g. cell spread area) and depth-dependent stiffness, among other factors^54^. To account for the inherently large variability of the mechanical properties of living cells, we probed a large number of cells for each neuronal type (between 30 and 50 cells in different cultures for each condition). The Young’s modulus values calculated for the different experimental conditions are compared in Fig. 6, with the mean values and standard errors of the mean summarized in Table 1.

For identical probing conditions, there were no significant differences in the elasticity of the membranes of untreated neurons (i.e. control) after 1 to 3 weeks in culture, including the aged preparation. As shown in Fig. 6, untreated neurons at different stages of aging and under different culture conditions have similar elastic modulus mean values, around 100 Pa, as calculated from the AFM force-indentation curves. However, the two Aβ oligomers interact differently with a given neuronal preparation. For the PNG-grown neurons, after 1 and 3 weeks, the membrane rigidity increased slightly in response to Aβ40 treatment compared to control, while Aβ42 treatment appeared to have the opposite effect by slightly softening the membrane. While the changes induced by Aβ40 were not significant, Aβ42 treatments induced more pronounced effects (*p* = 0.06 for 3 weeks PNG neurons and *p* < 0.0001 for aged neurons; Student t-test).

In the case of aged neurons, which exhibited enhanced aging hallmarks compared with young and mature cells (i.e. larger lipofuscin aggregates and decreased levels of cholesterol in the cellular membrane), both peptide species had a similar effect: a decrease in the stiffness of about 30%.

## Discussion

AFM data from primary rat hippocampal neurons show that membrane elasticity changes are induced by treatments with pre-aggregated Aβ species at sublethal concentrations. In particular, neurons grown in media deprived of neurotrophic factors display a fast and significant loss in membrane rigidity when exposed to both Aβ40 and Aβ42 oligomeric species in an early aggregation stage. The membrane elastic properties were probed by low force indentations to ensure the validity of the Hertz model.

These elasticity changes are correlated with the affinities of the Aβ species for the different neuronal preparations. Fluorescence microscopy indicates age-dependent differences in the ability of the Aβ oligomeric species to attach to the neuronal membrane. Both Aβ species bind more and with higher uniformity on mature and aged neurons, while a high variability in the binding affinity was observed for the young neurons. The coefficient of variation decreases significantly with the increasing neuronal age (see Table 1).

By investigating the mechanical properties of neurons treated with 2 hours pre-aggregated Aβ oligomers, we observed an interesting result. 3-week-old neurons cultured in a medium rich in vitamins and growth factors (i.e., PNG), although more susceptible to Aβ binding, had similar stiffness values to those of young neurons, with the Aβ42 treatment reducing more the elastic modulus of mature neurons. On the other hand, the Aβ40 seemed to induce a slight membrane stiffening in mature neurons, which could indicate a different interaction with the membrane, with Aβ40 potentially adsorbing at the membrane surface (leading to a stiffer structure), which is consistent with the proposed amyloid carpeting mechanism characteristic for amphipathic proteins^55^. On the other hand, Aβ42 may disrupt the lipid bilayer integrity via strong interactions with phospholipids^56^. However, when hippocampal neurons are aging under stress conditions (in our case, by culturing them in NBN2 medium), then they will show a strong decrease in elasticity after exposure to both Aβ40 and Aβ42 species. Earlier studies have shown that Aβ42 destabilizes lipid bilayers, as indicated by the reduction in critical lysis tension, thus resulting in an softening effect on giant unilamellar vesicles (GUVs)^13^,^57^. We detected that Aβ42 always reduced the stiffness of living primary neurons and the magnitude of this effect was correlated with lipid composition and the age of the neuronal preparation. Unexpectedly, we observed similar effects for Aβ40 in the case of aged neurons, which correlated with larger susceptibilities of the aged membranes to both Aβ isoforms (see Table 1). However, additional studies should be performed to link these results to the many suggestions that lipid metabolism plays a role in AD.

In a previous study investigating the effect of Aβ42 on N2a and HT22 cell lines^38^, the authors hypothesized that the ion influx triggered by Aβ42 treatment is responsible for the increased cellular rigidity through an increase in osmotic pressure. However, the increase in rigidity can also occur via strain-stiffening mechanisms in indented cells^58^ as cells respond to applied forces by increasing their elastic modulus^59^. In particular, when the applied forces are large, the stiffening is significant^60^. Thus, to avoid such dynamic responses, we performed the cellular indentations under weak applied forces that allow the study of cell surface effects under shallow deformations.

Mechanical forces are known to play an important role in processes such as gene expression, ion-channel gating and vesicular transport^41^. The mechanical properties of membranes define the way in which cells respond to such forces and therefore their ability to perform these functions. For instance, the active transport of vesicles in neurons is regulated by presynaptic membrane tension^40^, the activity of ion channels is dependent on membrane deformations^39^, and changes in the elasticity of the substantia nigra have been shown to occur in patients in the early stages of Parkinson’s Disease^61^. Although little is known about the mechanobiology of the brain^41^ one can expect that changes in the neuronal membrane elasticity or stiffness might directly induce functional changes related to neurodegeneration in AD, regardless of the mechanism underlying such changes.

## Materials and Methods

### Primary hippocampal neuron cultures

Primary hippocampal neurons were isolated from 18-day-old rat embryos (E18 embryos) (Wistar, Janvier). To obtain the embryonic hippocampal neurons, the animals were handled according to the international (EU Directive 86/609/EEC) and national laws governing the protection of animals used for experimental purposes, which minimize distress during procedures. The use of animals and the experimental procedures were approved by the Ethical Committee for Animal Welfare (ECD, Ethische commissie Dierenwelzijn) of the KU Leuven. The obtained neurons were plated on Poly-L-Lysine (Sigma-Aldrich, P2636-500MG)-coated 2.5 cm glass cover slips at a density of 10^5^ cells/well (6 wells plate) in Minimum Essential Medium (Invitrogen, 31095–029), glucose (Sigma-Aldrich, G7528-250G) and horse serum (Invitrogen, 31095–029) containing medium. After 3 hours, the medium was replaced with Primary Neuron Growth Medium (PNG - Lonza, CC-3256/CC-4462). The neurons were incubated at 37 °C, in 5% CO_2_ in air for 1 or 3 weeks in PNG to allow the hippocampal neurons to fully develop and ensure their long-term maintenance and growth. Aged neurons were obtained by gradually replacing with PNG medium with Neurobasal medium (NBN2 - Invitrogen, 21103–049) plus N2 supplement (Invitrogen, 17502–048) (1/3rd of medium per day), starting after 10 DIV. The NBN2 medium lacks antioxidants and vitamins, while still can maintain the viability of neurons. For the viability tests, 40.000 neurons were plated on PLL-coated 96-well plates, while for the preparation of the membrane pellets for the cholesterol content study, we plated 300.000 neurons per 60 mm dish.

### Aβ aggregation protocol

Solutions of either Aβ40 or Aβ42 (rPeptide, A-1153-1, A-1163-1) were prepared following a three-step protocol: first, the Aβ peptides were dissolved in 500 μl hexafluoroisopropanol (HFIP, Sigma-Aldrich, 105228-25G), a polar solvent that dissociates the pre-formed aggregates of Aβ. The HFIP was evaporated under nitrogen flow to prevent any risk of peptide oxidation. In the second step, the peptides were resolubilized in Dimethyl Sulfoxide (DMSO, Sigma-Aldrich, 41639) to maintain them in a dissociated state. Using a desalting column (GE-Healthcare, 17–1408–01), we obtained a solvent-free, monomeric solution of Aβ peptides in 50 mM Tris and 1 mM EDTA buffer (Trisaminomethane - Ethylene-Diamine-Tetraacetic Acid - Tris EDTA buffer). The final peptide concentration was measured using the Bradford assay (BioRad, 500–0005) with UV/visible absorption spectra acquired with an Ultrospec 2100 Pro spectrophotometer from GE Healthcare. The peptide solution was allowed to aggregate at room temperature (~25 °C) for 2 hours to ensure the presence of the peptide oligomers^8^,^43^,^62^. These oligomeric solutions were used for neuronal treatments in all experiments.

Next, 7 DIV or 21 DIV neurons in PNG medium and aged neurons (21 DIV in NBN2) were incubated with Aβ solutions at a final concentration of 10 μM in Tris EDTA buffer for at least 1 hour before performing the AFM measurements.

### Viability test

The CellTiter-Blue^®^ Cell Viability Assay (Promega) was used to evaluate potential viability changes of control and Aβ-treated neurons. The three neuronal preparations were treated with 10 μM Aβ oligomers for 3 hours and then incubated with CellTiter-Blue reagent for 2.5 hours. A Tecan spectrophotometer was employed to measure the fluorescence of CellTiter-Blue^®^ Cell Viability Assay.

### Fluorescence imaging

Cells were fixed for 30 minutes in a solution containing 4% paraformaldehyde with 4% sucrose in Dulbecco’s Phosphate-Buffered Saline (DPBS) followed by a three-step DPBS (Invitrogen, 14190–094) rinsing protocol. The fixed neurons were maintained in DPBS at 4 °C until further measurements.

Lipofuscin is an autofluorescent lipopigment that is correlated with aging of non-mitotic cells, such as neurons or cardiomyocytes. Autofluorescence in UV light is a feature of this pigment. An excitation wavelength of 405 nm was used, and emission was measured with a band filter between 505–605 nm. Lipofuscin imaging was performed on fixed neurons in DBPS using a FV1000 Confocal Laser Scanning Microscope from Olympus. The image processing was performed in ImageJ.

To confirm the Aβ susceptibility of the neuronal membrane, the cells were treated for 3 hours with 10 μM Aβ oligomers prepared as described. After fixation, the neurons were labeled with the anti-Aβ fluorescent primary antibody, Beta Amyloid 1–16 (6E10) Monoclonal Antibody, and Alexa Fluor 488 Labeled (Eurogentec) at a dilution of 1:500 for 2 hours. Aβ-labeled neurons were maintained in DPBS at +4 °C until further measurements.

Epifluorescence imaging of living or fixed cells was performed with an Orca-Flash 4.0 Hamamatsu camera connected to an IX81 Olympus Microscope. The image processing was performed in ImageJ.

### Cholesterol levels in the neuronal membrane

Membrane pellets were obtained from neurons cultured in 60-mm petri dishes. The cells were lysed in MES buffer (25 mM, pH 7.1), and the membrane pellet of the neurons was obtained by ultracentrifugation of the extract at 70000 rpm (TLA 100.1 rotor, Beckman) for 1 hour at 4 °C. Quantification of cholesterol concentration was performed using the Amplex Red cholesterol assay (Invitrogen- A12216). Fluorescence levels in the final AmplexRed–membrane pellet solution were measured using a Tecan spectrophotometer.

### AFM studies

AFM experiments were performed on a NanoWizard 3 BioScience AFM (JPK Instruments, Germany) integrated in an iX81 microscope frame (Olympus, Belgium). The Biocell sample holder (JPK) maintains the cells at physiological temperature conditions during the measurements.

High-resolution AFM topographical images of Aβ oligomers at different aggregation stages were taken using a sharp silicon oxide tip attached to a very soft V shape silicon nitride cantilever (MSNL-10, cantilever F, Bruker: spring constant 0.5 N/m and resonant frequency 90–160 kHz), while fixed neurons were imaged with a softer cantilever tip (MSNL-10, cantilever C, Bruker: initial radius between 2 and 12 nm, nominal spring constant 0.01 N/m and resonant frequency 4–10 kHz). A SEM micrograph of the imaging tip is shown in Fig. S4A - Supplementary Information. All AFM tests on living neurons were performed under constant temperature conditions, and each experimental session was limited to 2 hours.

Force spectroscopy was performed using the Quantitative Imaging mode (QI) of the JPK system (QI-JPK). The fixed neurons were imaged in QI mode at a resolution of 256 × 256 pixels using the sharp cantilever with a set point of 2 nN and 70 μm/s speed, and the scanned surface area of 30 × 30 μm^2^.

The spring constant of the cantilever was measured in medium at 37 °C and calculated before every experiment using the JPK software Calibration Manager.

### AFM elasticity measurements

For the elasticity measurements, we used spherical tips, i.e.15 μm polystyrene beads, to ensure complete cell body indentation and reduced stress localization on the cellular membrane. The bead was attached on the MSNL-10, cantilever C, Bruker cantilever with Paxitol glue (a scanning electron micrograph is shown in Fig. S4B – see Supplementary information). The attachment of the sphere neither modified the spring constant nor the sensitivity of the cantilever. The Young’s modulus of the glued bead was 100 kPa, 10^3^ times higher than neuronal modulus.

The Force-spectroscopy JPK acquisition mode was used. The neuronal cell body was indented by the 15 μm polystyrene sphere attached to the soft cantilever using different forces (0.2, 0.5, 1, 2, 3, 4 nN) at a ramping speed of 5 μm/s with a ramp frequency of 2 Hz and a vertical ramp size of 5 μm (see Fig. S2 - Supplementary Information). Force-distance curves were further processed using Data Processing JPK software to extract the indentation depth and elastic properties. The Young’s modulus was calculated from the approaching part of the force-indentation curves using the force-indentation relationship described in the Hertz model for the spherical indenter, as described by the equation (1) 63:


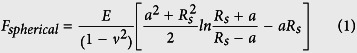


with


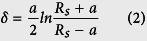


where *F*_*spherical*_ is the indentation force, *E* is the Young’s modulus, 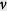 is Poisson ratio, 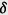 is the indentation depth, *a* the radius of the contact circle, and *R*_*s*_ the radius of the spherical indenter. We assumed a Poisson ration of 0.46 (specific for hippocampal neuron body^64^).

### Spontaneous activity recording by MEA

Neurons were plated at 1000 cells mm^−2^ on a MEA substrate (Multichannel Systems GmbH, Germany) and grown for 8–10 days. The spontaneous firing rate of the neuronal network was recorded simultaneously from 30 to 50 signal collecting electrodes (out of 60 available)^65^,^66^. During the recording experiment, a temperature controller from Multichannel Systems was used to maintain the MEA platform temperature at 37 °C. The basal firing rate was recorded during 120 s. Upon treatment with Aβ, the spontaneous activity was continuously recorded for up to 40 minutes. Raw signals from MEA electrodes were amplified by MEA1060 amplifier (gain 1200) from Multichannel Systems and digitized by the A/D MC_Card at a sampling rate of 25 kHz. MC_Rack 3.5.10 software (Multichannel Systems) was used for data recording and processing. The raw data stream was high‐pass filtered at 200 Hz, and the threshold for spike detection was set to 5 SD of the average noise amplitude computed during the first 1000 ms of recording. The number of spikes detected from every electrode per time bin of 120 s was normalized to baseline (firing rate in the absence of treatment). After data analysis, the firing rates after 5, 20 and 30 minutes of treatment were extracted and presented as percentage of initial rate.

## Additional Information

**How to cite this article**: Ungureanu, A.-A. *et al.* Amyloid beta oligomers induce neuronal elasticity changes in age-dependent manner: a force spectroscopy study on living hippocampal neurons. *Sci. Rep.* **6**, 25841; doi: 10.1038/srep25841 (2016).

## Supplementary Material

Supplementary Information

## Figures and Tables

**Table 1:** Susceptibilities of different neuronal preparations to the binding of pre-aggregated Aβ40 and Aβ42 peptides and the elastic moduli recorded (set point = 0.2 nN).

Neuron age/type and Aβ treatment	Mean percentage of Aβ-positive (fluorescent) neurons	Coefficient of variation (CV)	Total number of neurons (bright field images)	Mean Young modulus (Pa) for shallow indentations (0.2 nN)	Std. error of the mean
1w PNG control	**–**	**–**	**–**	116.46	9.76
1w PNG + Aβ40	34%	73%	189	110.98	8.38
1w PNG + Aβ42	52%	29%	223	106.96	13.61
3w PNG control	**–**			103.91	7.60
3w PNG + Aβ40	82%	20%	264	112.64	11.01
3w PNG + Aβ42	79.5%	18.5%	173	84.41***	9.44
3w PNG/NBN2	**–**			112.37	6.91
3w PNG/NBN2 + Aβ40	97.7%	5%	296	72.94*	6.00
3w PNG/NBN2 + Aβ42	89.7%	11%	346	70.95**	6.74

*p, **p < 0.0001, ***p = 0.06 (unpaired t-test).
