# Supplementary material for: Amyloid beta oligomers induce neuronal elasticity changes in age-dependent manner: a force spectroscopy study on living hippocampal neurons
Source: Sci Rep. 2016 May 13;6:25841. doi: 10.1038/srep25841 (PMC4865860; doi:10.1038/srep25841)
Supplement: Supplementary Information [file srep25841-s1.pdf]

# Amyloid beta oligomers induce elasticity changes in the neuronal membrane in an age-dependent manner: a force spectroscopy study on living primary hippocampal neurons

Andreea-Alexandra Ungureanu*[1,2], Iryna Benilova[3], Olga Krylychkina[2], Dries Braeken[2], Bart De Strooper[3], Chris Van Haesendonck[1], Carlos G. Dotti[3,4], Carmen Bartic[1,2]

**Affiliations**

[1] Department of Physics and Astronomy, KU Leuven, Celestijnenlaan 200D, B-3001, Leuven, Belgium

[2] IMEC, Kapeldreef 75, B-3001 Leuven, Belgium

[3] VIB Center for the Biology of Diseases, ON 4 Campus Gasthuisberg, Herestraat 49, B-3001, Leuven, Belgium

[4] CSIC, Centro de Biología Molecular Severo Ochoa, Universidad Autónoma de Madrid, Campus Cantoblanco, 28049 Madrid, Spain

## Supplementary figures

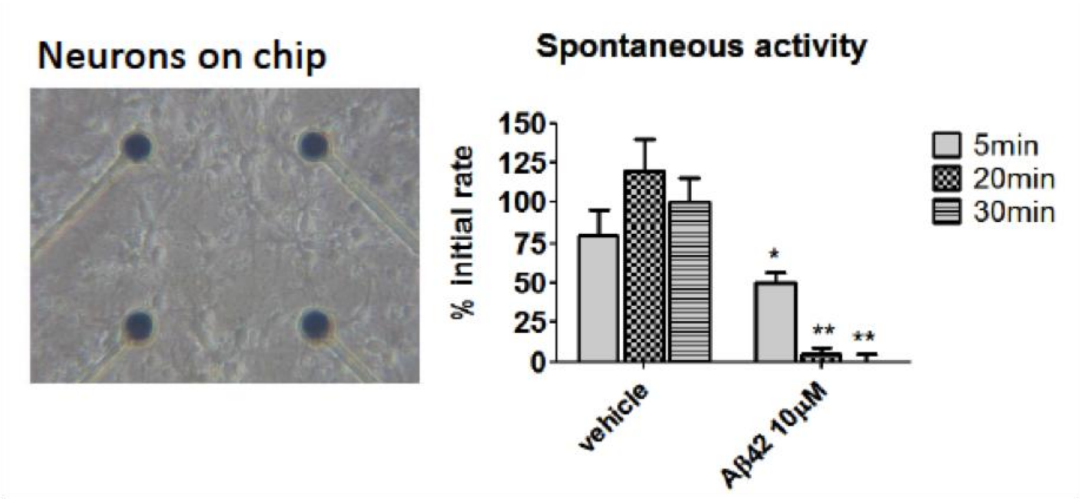


**Figure S1.** *(Left)* Neuronal culture on a MEA chip; *(Right)* 10uM of Aβ42 pre-aggregated for 2h acutely inhibits spontaneous electrical activity of primary hippocampal neurons on MEA (**p* < 0.05, ***p* < 0.01, Student's t-test, n = 30 - 50 active electrodes from three independent chips). Firing rate was measured after 5, 20 and 30 minutes respectively.

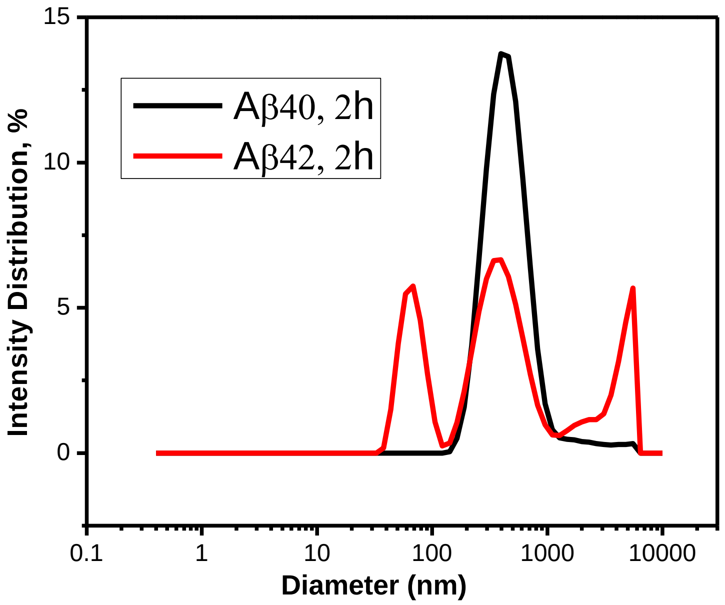


**Figure S2.** Dynamic light scattering (DLS) measurements of the Aβ40 and Aβ42 oligomeric species present in solution after 2 hours of aggregation in Tris EDTA buffer at RT. DLS is detecting also the larger species and reveals a more heterogeneous size distribution for Aβ42 as compared to Aβ40. Smallest aggregate sizes are detected for Aβ42.

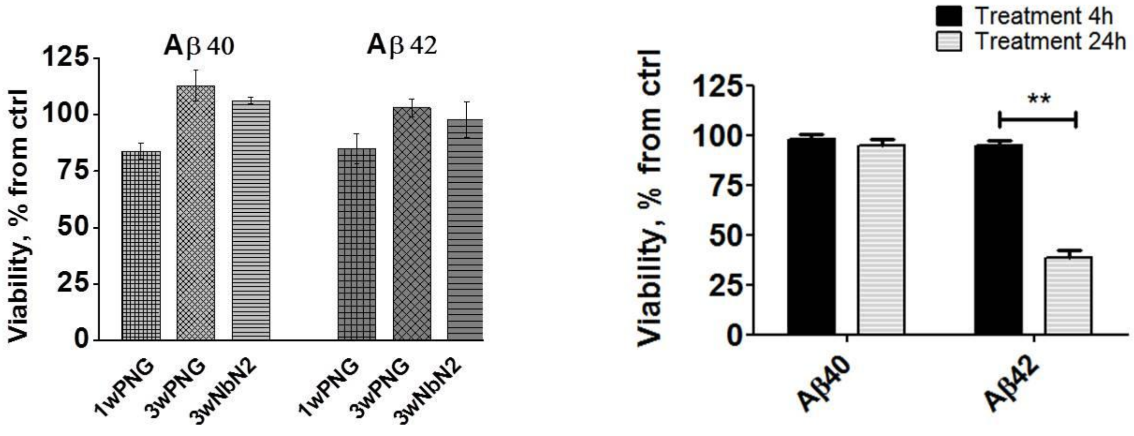


**Figure S3.** *(Left)* No significant viability changes were detected after a treatment of 3 hours with 10μM solutions of Aβ40 and Aβ42 solutions; *(Right)* After a 24h treatment with Aβ42 the viability drops by approximately 60% (CellTiter-Blue® Cell Viability Assay).

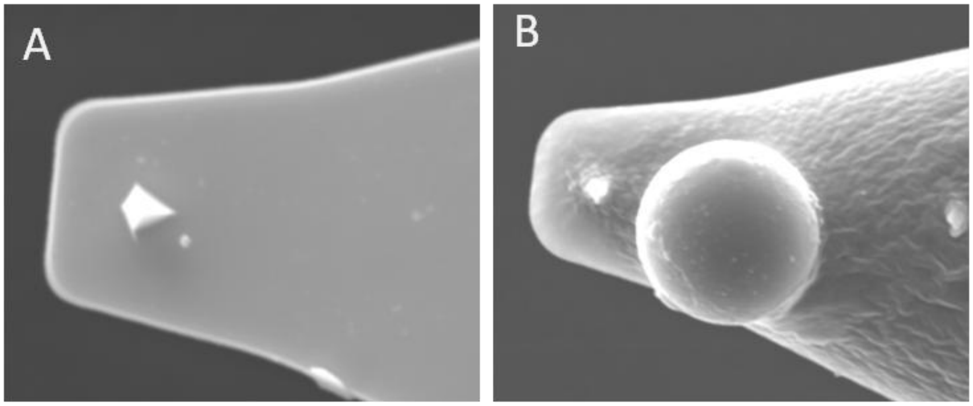


**Figure S4.** SEM images of a bare MSNL-C probe (A) and a MSNL-C probe modified with a 15 μm Polystyrene sphere. The sharp MSNL-C probe was used for obtaining detailed topographical images of neuronal cells. For the elasticity measurements the MSNL-C probe was modify with a 15 μm polystyrene sphere to assure the complete cell soma compression and to avoid damaging the cell membrane. The bead was attached on the soft MSNL-C cantilever (spring constant of 0.01 N/m). The attachment of the sphere does not modify the spring constant or the sensitivity of the cantilever.

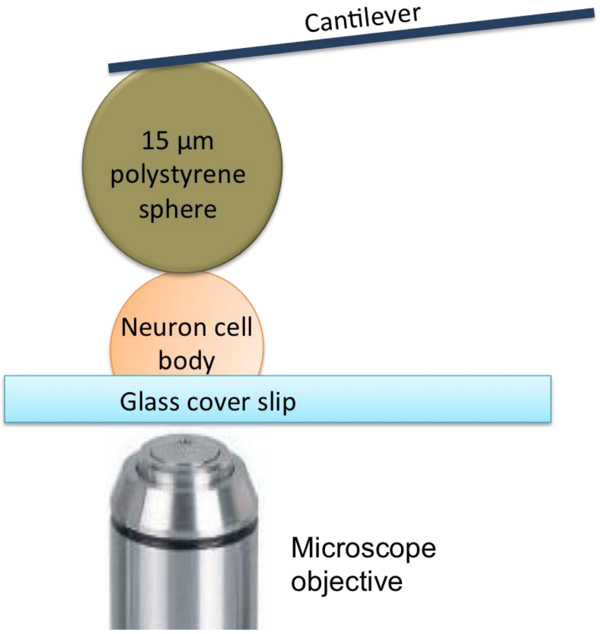


**Figure S5.** Elasticity measurement setup illustration: The AFM device is integrated in an optical microscope (Olympus IX81) that allows the overlay of the AFM scan with the optical image.
